# Enhancing Mechanical and Thermal Properties of 3D-Printed Samples Using Mica-Epoxy Acrylate Resin Composites—Via Digital Light Processing (DLP)

**DOI:** 10.3390/polym16081148

**Published:** 2024-04-19

**Authors:** Velmurugan Senthooran, Zixiang Weng, Lixin Wu

**Affiliations:** 1 CAS Key Laboratory of Design and Assembly of Functional Nanostructures, Fujian Key Laboratory of Nanomaterials, Fujian Institute of Research on the Structure of Matter, Chinese Academy of Sciences, Fuzhou 350002, China; velmurugan@fjirsm.ac.cn (V.S.); wzx@fjirsm.ac.cn (Z.W.); 2 University of Chinese Academy of Sciences, Beijing 100049, China

**Keywords:** digital light processing, mica powders, epoxy acrylate resin, 3D-printing

## Abstract

Digital light processing (DLP) techniques are widely employed in various engineering and design fields, particularly additive manufacturing. Acrylate resins utilized in DLP processes are well known for their versatility, which enables the production of defect-free 3D-printed products with excellent mechanical properties. This study aims to improve the mechanical and thermal properties of 3D-printed samples by incorporating mica as an inorganic filler at different concentrations (5%, 10%, and 15%) and optimizing the dispersion by adding a KH570 silane coupling agent. In this study, mica was introduced as a filler and combined with epoxy acrylate resin to fabricate a 3D-printed sample. Varying concentrations of mica (5%, 10%, and 15% *w*/*w*) were mixed with the epoxy acrylate resin at a concentration of 10%, demonstrating a tensile strength increase of 85% and a flexural strength increase of 132%. Additionally, thermal characteristics were analyzed using thermogravimetric analysis (TGA), and successful morphological investigations were conducted using scanning electron microscopy (SEM). Digital light-processing technology was selected for its printing accuracy and cost-effectiveness. The results encompass comprehensive studies of the mechanical, thermal, and morphological aspects that contribute to the advancement of additive manufacturing technology.

## 1. Introduction

The field of 3D printing technology has witnessed rapid advancements in recent years, transforming the landscape of manufacturing and design [1,2,3,4]. Researchers are continually pushing the boundaries of 3D printing capabilities and exploring new materials, techniques, and applications [1,2,3]. One area of focus involves the development of sustainable and biodegradable printing materials to address environmental concerns [5]. Recent research articles have developed novel bio-based polymers and composite materials that not only offer enhanced printability but also align with the growing demand for eco-friendly manufacturing processes [6,7,8,9,10]. Furthermore, there is a notable emphasis on improving the precision and speed of 3D printing through innovations such as high-speed resin-based printing and advanced multi-material printing technologies [11,12,13,14,15]. The integration of artificial intelligence and machine learning algorithms to optimize print parameters and minimize defects is another exciting avenue explored in recent studies. These research endeavors collectively contribute to the evolution of 3D printing as a versatile and sustainable manufacturing method with implications for industries ranging from healthcare and aerospace to consumer goods and construction [1,2,3,16,17,18].

Common constituents of mica, a clay mineral, include potassium (K), aluminum (Al), silicon (Si), oxygen (O), fluorine (F), and hydroxyl (OH) ions [1,2]. The tiny, flexible layers of mica flakes contribute to the exceptional cleavage and distinctive quality of the material When applied as a filler to epoxy resin during the vat polymerization process, mica provides reinforcements and enhances the mechanical properties of materials due to its high aspect ratio [15]. Ideal for electrical and electronic applications, such as insulators, capacitors, and circuit boards, mica exhibits high electrical strength, low power loss, and low capacitance [19]. Their remarkable heat resistance makes mica suitable for fire-resistant applications, even though it is sensitive to strong acids and alkalis. This chemical resistance makes it suitable for environments where such resistance is required [19,20,21,22]. Depending on the processing method, mica can be either transparent or opaque. The strategic incorporation of mica as a filler during vat polymerization enhances the mechanical strength of the epoxy resin, with the mineral’s unique platy structure ensuring no need to remove its uniform distribution within the resin matrix [19,20,21]. This constructive collaboration not only improves the overall mechanical performance but also opens avenues for applications in industries requiring robust materials, such as automotive components, aerospace structures, and advanced engineering materials. The use of mica in epoxy resins during vat polymerization has the potential to elevate material properties, offering a promising pathway for developing high-performance composite materials across diverse industrial applications [23,24,25,26,27,28,29].

Epoxy acrylate resins are extensively employed in digital light processing (3D printing) techniques because of their exceptional attributes, including corrosion resistance; excellent electrical insulation; high tensile, flexural, and compressive strength; thermal stability; and resistance to chemicals and corrosion [19,20,21,22,23]. Mica has been used as filler in epoxy acrylate composites in various applications. Some recent research studies demonstrate the potential of reduced mica particle size to enhance epoxy acrylate/mica composite coatings, and they also show the optimal particle size for maximal performance enhancement [18,30,31,32]. Some studies showcase enhancements in mechanical and thermal properties with nanoclay addition to DLP 3D-printed parts, suggesting a broader research gap in pinpointing the mechanisms behind these improvements and how they might be maximized with varying nanoclay concentrations [19,20,21,22,23,24,29]. These gaps collectively emphasize the need for further research into the intricate balance between additive types, concentrations, and resulting material properties to advance the capabilities of DLP 3D printing technology in achieving superior mechanical and thermal performance in mica and epoxy acrylate-reinforced composites [1,23,24]. Continued investigation into the synergistic effects of additive types and concentrations holds promise for unlocking the full potential of mica and epoxy acrylate-reinforced composites in DLP 3D printing, driving innovation and advancements in material performance and application versatility [26,27,28,29].

This will provide an area for the open exploration of inorganic-filled epoxy acrylate resin for 3D-printed parts. In this study, the incorporation of mica as a filler into epoxy acrylate was explored using DLP 3D printing, facilitated by the addition of a silane coupling agent (KH570) for even dispersion in the acrylate resin. This approach aims to achieve optimal mechanical and thermal properties, along with a thorough investigation of the morphological characteristics. The outcomes demonstrated favorable mechanical properties, particularly notable improvements in the tensile and flexural strength percentages. The applications of this research hold promise in various fields, including the development of thermal insulators and engineering products, highlighting the potential for the enhanced performance of 3D-printed materials.

## 2. Materials and Methods

### 2.1. Materials

Mica powders with an average size of 25 µm were procured from Henan, China. The epoxy acrylate resin formulations consisted of urethane acrylate (CN991), bisphenol-A epoxy acrylate (CN104S), pentaerythritol triacrylate (SR444NS), tri (propylene glycol) diacrylate, ethoxylated trimethylolpropane triacrylate (SR306NS), acryloylmorpholine (ACMO), and diphenyl (2,4,6-trimethylbenzoyl) phosphine oxide (TPO), all sourced from Sartomer America (Exton, PA, USA). The silane coupling agent KH570 and Isopropyl Alcohol (IPA) were obtained from the Aladdin Chemical Company (Ontario, CA, USA).

### 2.2. Preparations Methods of UV-Curable Epoxy Acrylate and Mica Mixer Resin

**Figure 1:**
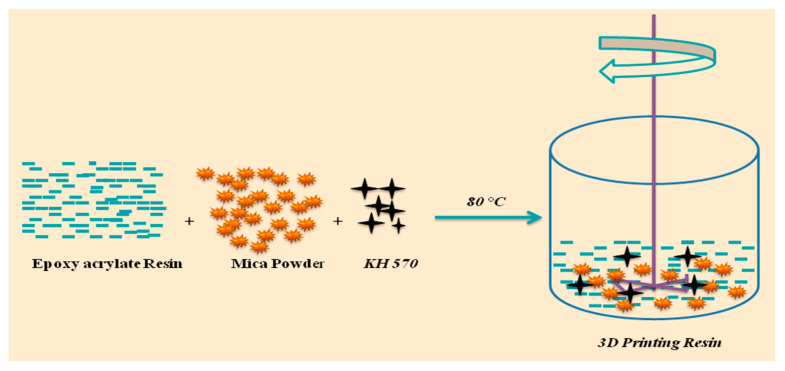
Mixing of mica and EPA resin with the addition of KH570 at 80 °C.

Initially, a UV-curable epoxy acrylate resin was prepared by mixing monomers and reactive dilutants, as listed in Table 1. The resin was homogenously mixed using a homogenizer. The experimental setup is shown in Figure 1. In this procedure, 1 g of KH570 was blended with deionized water and ethanol at a weight ratio of 1:1:20. Following 5-minute hydroxylation at room temperature, the resulting KH570 solution was gradually introduced into a mixture of epoxy acrylate resin and mica dispersion. The mixture was stirred for 1 h at 80 °C. Subsequently, the epoxy acrylate resin and mica powder were accurately weighed and calculated in terms of weight percentage ratios of EPA, EPA/mica 5%, EPA/mica 10%, and EPA/mica 15%, respectively, with the addition of KH570 to ensure proper dispersion.

### 2.3. 3D Printing Using a DLP

**Figure 2:**
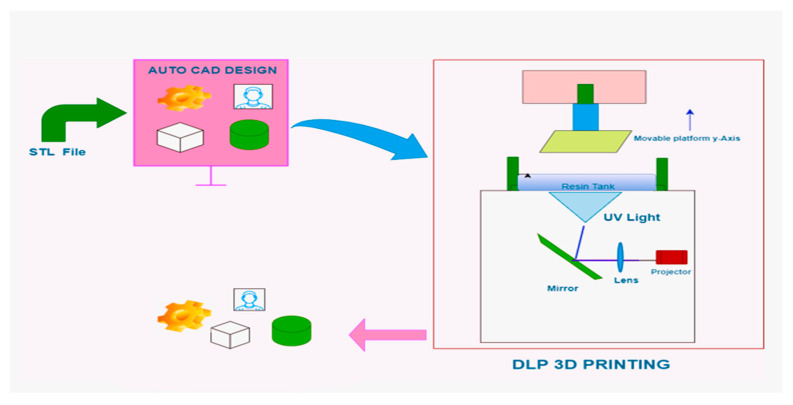
Overview of the 3D printing of mica-filled epoxy resins using DLP.

In this study, UV-curable resins were utilized in a digital light processing (DLP) setup, employing a projector from Zhejiang Xunshi Technology Co., Ltd., Shaoxing, China, emitting light at a wavelength of 405 nm with a power of 3 mW/cm^2^, as illustrated in Figure 2. To ensure adherence to standardized testing procedures, all samples were prepared according to the American Society for Testing and Materials [ASTM Type-4 D638, [33]]. During the 3D printing process, printer parameters were meticulously optimized, with particular attention being paid to the bottom exposure time (8.5 s) and curing rate (5 s), as detailed in Table 2. This optimization process aimed to achieve optimal print quality and material properties. The dimensional accuracy of the fabricated samples was meticulously in conjunction with Computer-Aided Design (CAD) models, ensuring the precise and consistent printing of well-defined structures. Following printing, any excess uncured resin present on the printed parts, referred to as “green bodies”, was thoroughly cleaned using isopropanol (IPA), ensuring the removal of any residual material and resulting in the production of high-quality, defect-free samples suitable for subsequent analysis and testing in Figure 13.

### 2.4. Testing and Characterization

The analysis of EPA (epoxy acrylate resin) and EPA/mica (5, 10, and 15 wt.%) samples involved using an XRD (Mini flex 600, Rigaku, Tokyo, Japan), with Cu-Kα radiation (λ = 1.59041 Å), within a 10–80° angle range. The results revealed structural changes in EPA after the addition of mica as a filler. FTIR Thermo Fisher Scientific equipment (Thermo Fisher Scientific, Waltham, MA, USA) was employed to validate the IR spectra of EPA (epoxy acrylate resin) and EPA/mica. The average scanning rate was 16 scans, and the range varied from 4000 cm^−1^ to 400 cm^−1^. Mechanical properties, such as tensile and flexural properties, were determined using a universal material testing machine (AGX-100Plus, Shimadzu, Kyoto, Japan). Tensile testing was performed according to [Type-4, ASTMD638] standard specimen type 4. Flexural properties were measured using the [ASTMD 790, [34]] three-point bending method. The density of the materials was measured using an ASTMD 792 [35] density tester (Unlong DX-300X, Huish Outdoors, LLC, Salt Lake City, UT, USA). Thermogravimetric analysis (TGA, TGA-101, Shanghai Hesheng Instrument, Shanghai, China) involved heating the samples from 0 °C to 600 °C at a rate of 10 °C/min under a nitrogen atmosphere. Additionally, the morphologies of EPA and mica-reinforced EPA 3D-printed samples were observed using a Field Emission Scanning Electron Microscope [FESEM) [JSM-7500F, SU8010/EDX, JEOL Ltd., Tokyo, Japan) following the tensile study analysis.

## 3. Results and Discussion

### 3.1. Dispersion Study

**Figure 3:**
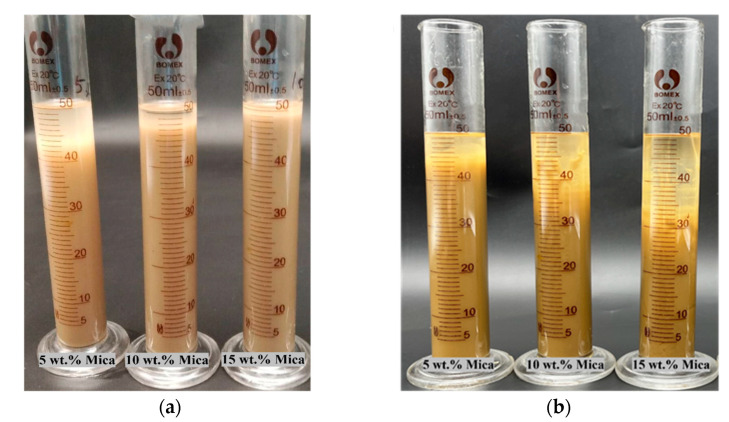
(**a**) EPA/mica with 1 g of KH570 silane coupling agent, and (**b**) EPA/mica without a coupling agent.

The dispersion of mica fillers into epoxy acrylate resin is a difficult process, and even if it is dispersed mechanically for long-term stability, it is not as good as we need. Mica fillers settle during the printing process or are unevenly distributed in the resin matrix. To overcome this issue, the filler surfaces were modified using coupling agents. These coupling agents are used to couple inorganic fillers to organic resin materials to achieve proper dispersion and long-term stability. This was also maintained through an appropriate particle size. Here, we used a silane coupling agent to modify mica particles and couple them with the resin matrix. This resulted in an even dispersion of mica particles in the resin, which was maintained for a long time. Figure 3 shows the difference before and after the 20-day gap, which clearly explains the dispersions. Figure 3b shows EPA/mica ((5, 10, 15) *w*/*w*%) without KH570. In addition, Figure 3a shows EPA/mica ((15, 10, 5) *w*/*w*%) with the addition of the silane coupling agent KH570. In composite materials, silane coupling agents improve the compatibility and dispersion of mica fillers and epoxy acrylate resins. This study identified the proper dispersion of mica particles in epoxy acrylate resin caused by the addition of 1 g of KH570.

### 3.2. XRD Analysis

**Figure 4:**
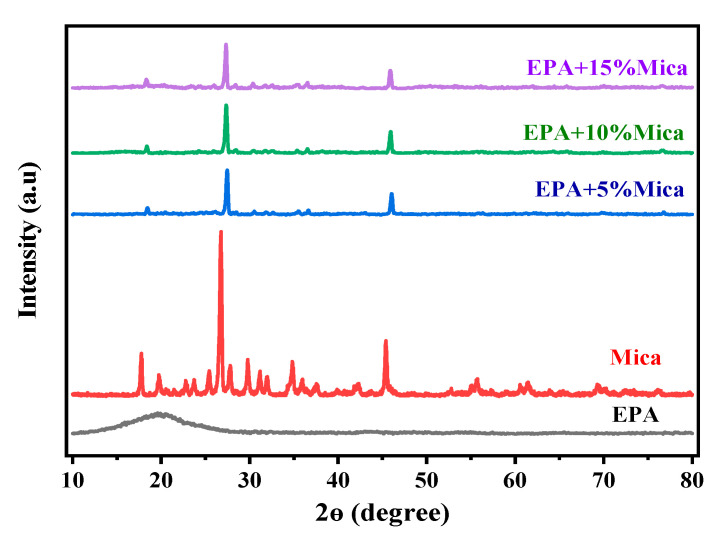
The X-ray diffraction results of the base EPA and EPA/mica 5, 10, and 15 wt.% concentrations.

The X-ray diffraction (XRD) study of the epoxy acrylate resin (EPA), i.e., the addition of less than 15% mica, revealed significant alterations in the crystalline structure, as shown in Figure 4. The diffraction patterns of the modified resin changed, indicating possible interactions between the diffraction patterns of the resin matrix. The mica diffraction pattern and mica with epoxy acrylate resin showed changes in the peak intensities and shifts in the peak positions. The peak positions and intensities shifted, indicating changes in the arrangement and packing of molecular entities within the resin. This is because the epoxy resin is amorphous in structure, and the mixing of amorphously structured resin with mica causes shifts in positions and changes in intensities. These data indicate that the addition of mica altered the crystallinity of the epoxy acrylate resin, thereby affecting its overall structural properties [2]. The XRD results provide vital insights into nanoscale structural changes, opening the way for a deeper understanding of the improved characteristics and performance of mica-modified epoxy acrylate resins, which is relevant to a variety of industrial applications. This XRD peak indicates that the crystal grains were contaminated with impurities during reinforcement.

### 3.3. Fourier Transformed Infrared Spectroscopy

**Figure 5:**
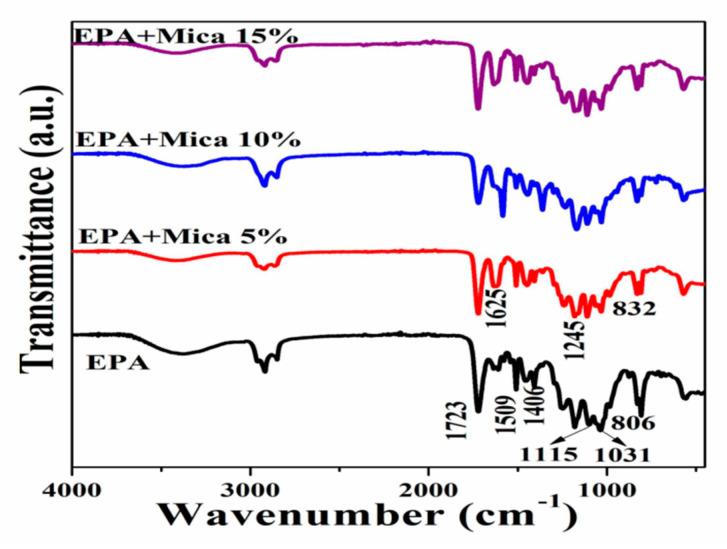
FTIR spectra of base EPA and EPA/mica (5, 10, 15) % concentrations.

Fourier transform infrared spectroscopy (ATR) mode examination of the epoxy acrylate resin, both before and after adding less than 15% mica, indicated significant changes in the functional peaks, as illustrated in Figure 5. The signal at 1723 cm^−1^, which corresponds to the CO stretching of acrylic acid, shifted in the modified resin, indicating probable interactions between mica and the acrylic acid moiety. Variations in the aromatic ring peaks were also identified, with shifts at 1625 cm^−1^ and 1509 cm^−1^ corresponding to C=C and C-C stretching, respectively. The shear vibration absorption peak of -CH2 at 1408 cm^−1^ changed, indicating potential alterations in the molecular structure of the resin. Furthermore, shifts in the peaks linked to the epoxy group were detected, such as the C-O-C ether stretching at 1031 cm^−1^ and the epoxy C-O stretching at 983 cm^−1^, indicating interactions between the epoxy resin and mica [3]. These FTIR results provide useful insights into the chemical changes caused by the addition of mica to epoxy acrylate resin, which can have good performance and applications.

### 3.4. Tensile Properties

**Figure 6:**
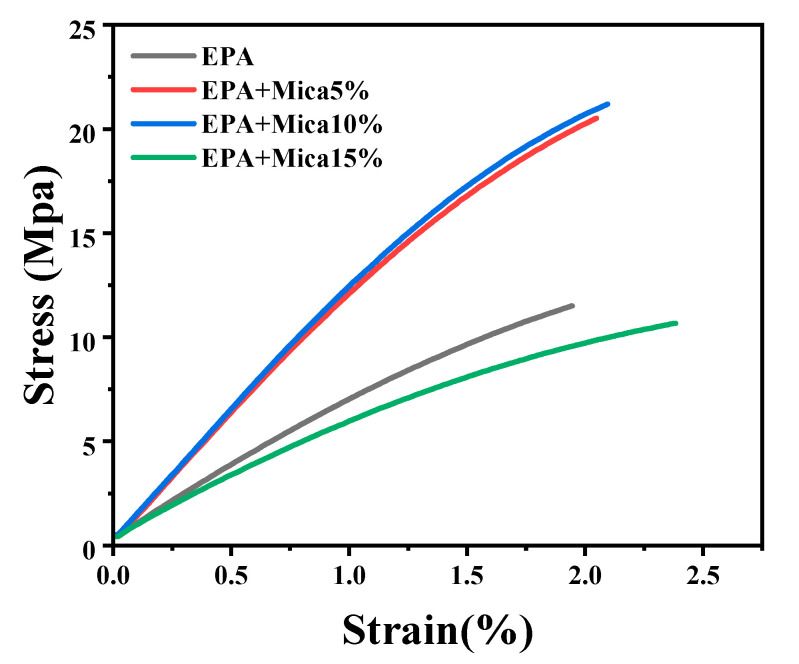
Stress–strain curves of EPA resin and EPA/mica at different weight ratios.

Stress–strain assessments of the epoxy acrylate resin with and without the addition of less than 15 wt.% mica demonstrated considerable mechanical improvements, as shown in Figure 6. The inclusion of mica increased the tensile strength of the resin, as indicated by the stress–strain curves. The addition of 10% mica powder reinforced the resin matrix, increasing the tensile strength and load-bearing capability compared with the other samples. The tensile strengths of the 5 wt.% and 10 wt.% mica-loaded samples increased by 50% compared with pure epoxy acrylate resin. However, the 15 wt.% mica-loaded composite material has lower tensile strength compared with epoxy acrylate resin. This behavior may have occurred because of the higher loading percentage of mica. This may be because the crosslinking of the epoxy acrylate resin was reduced through the interactions of the mica particles during the 3D printing process. The elongation of the 15 wt.% mica-loaded composite increased because of these changes, but its tensile strength was lower than that of the pure epoxy acrylate resin. These changes in mechanical behavior show that the mica-modified epoxy acrylate resin has a more robust and enduring performance, making it ideal for applications that require a high mechanical strength. Furthermore, the 15% mica-modified epoxy acrylate resin showed a slightly reduced tensile strength, confirming that a higher mica content leads to a lower tensile property. The stress and strain study provided essential data for understanding the structural integrity and mechanical enhancements caused by the integration of 10% mica in the epoxy acrylate resin, which contributed to the development of higher performance.

### 3.5. Flexural Properties

**Figure 7:**
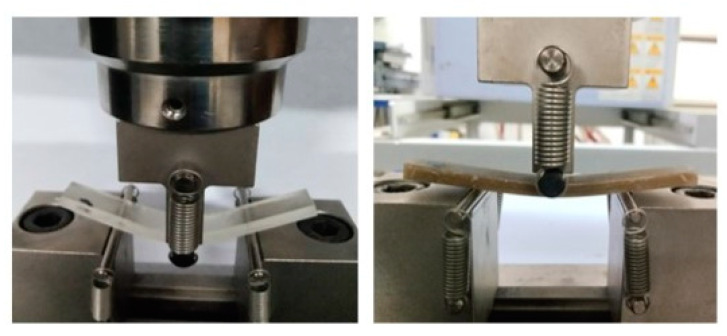
Flexural three-point bending test.

**Figure 8:**
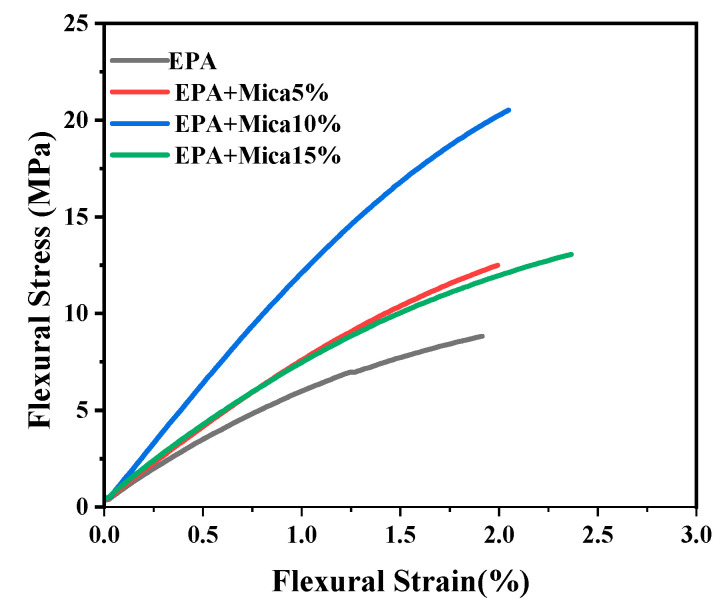
Flexural strain versus flexural stress of base EPA and EPA/mica ((5, 10, and 15) *w*/*w*%) concentrations.

A mica content of less than 15% significantly increased the bending resistance of the material. The addition of 10% mica significantly enhanced the flexural strength by 132%, indicating that the resin could enhance the flexural strength better than 5 wt.% and 15 wt.% mica-modified epoxy acrylate resins in Figure 7. The observed changes in the flexural strength underscore the reinforcing effect of mica within the resin matrix, resulting in a more resilient and durable composite material in Figure 8. These findings have practical significance for applications that require materials with high bending strengths, implying that the 10% mica-modified epoxy acrylate resin has the potential for use in structural components and other load-bearing applications where strong mechanical performance is critical. Flexural strength research provides useful insights into the mechanical advantages of mica inclusions, indicating the potential applicability of modified resins in a variety of technical and industrial situations.

**Figure 9:**
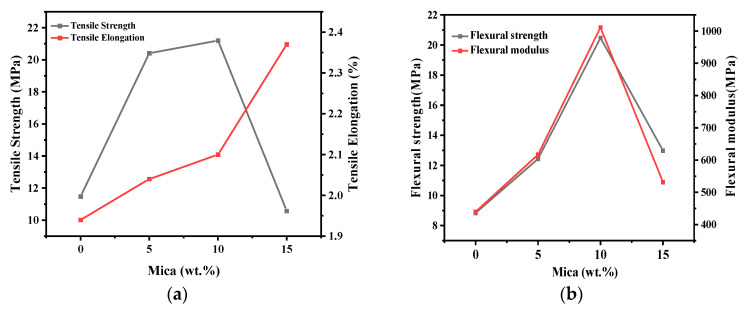
(**a**) Comparative data of tensile strength and tensile elongation of the composite material, and (**b**) comparative data of flexural strength and tensile flexural modulus.

The overall changes in the mechanical properties of the composite materials with the addition of mica at various contents of wt.% are given in Table 3. Figure 9a shows comparative data regarding the tensile properties of the composite materials. The data show that the tensile strength of the EPA/mica composites increased up to 10 wt.% and suddenly decreased at 15 wt.%. In the case of the tensile elongation, the composites exhibited an increasing trend from 0 to 15 wt.%. We ensured that the density changes owing to the increase in filler content were measured using the immersion method. The results suggest that the dispersion of the mica filler was even for all three composites. The increases in density indicate the presence of mica fillers in the printed composites. Simultaneously, the flexural strength and flexural modulus data in Figure 9b exhibit an increasing trend from 0 wt.% to 10 wt.% and a decrease at 15 wt.%.

### 3.6. Thermo Gravimetric Analysis

**Figure 10:**
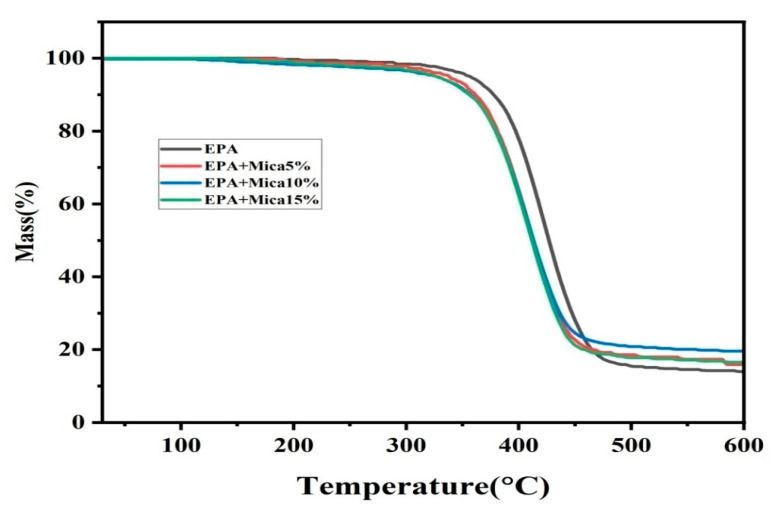
The thermogravimetric images of the base EPA and EPA/mica ((5, 10, and 15) *w*/*w*%) concentrations.

Thermogravimetric analysis of the epoxy acrylate resin before and after adding mica demonstrated significant changes in the thermal stability of the composite material. The Figure 10 shows TGA curves show variations in the weight loss patterns, indicating altered thermal degradation properties. The additions of mica as 5, 10, and 15 wt.% fillers show increasing thermal stability compared to the pure epoxy acrylate resin. The inclusion of mica improved the thermal stability of the resin, as demonstrated by the changes in the initial decomposition temperature and rate of weight loss at higher temperatures. These findings indicate that mica-modified epoxy acrylate resin is more resistant to high-temperature conditions, making it potentially suitable for applications that require better thermal performance. Thermal information on the thermal behavior of a composite material is crucial for determining its applicability in various industrial and technological fields, where thermal stability is a critical issue.

### 3.7. Morphological Study

**Figure 11:**
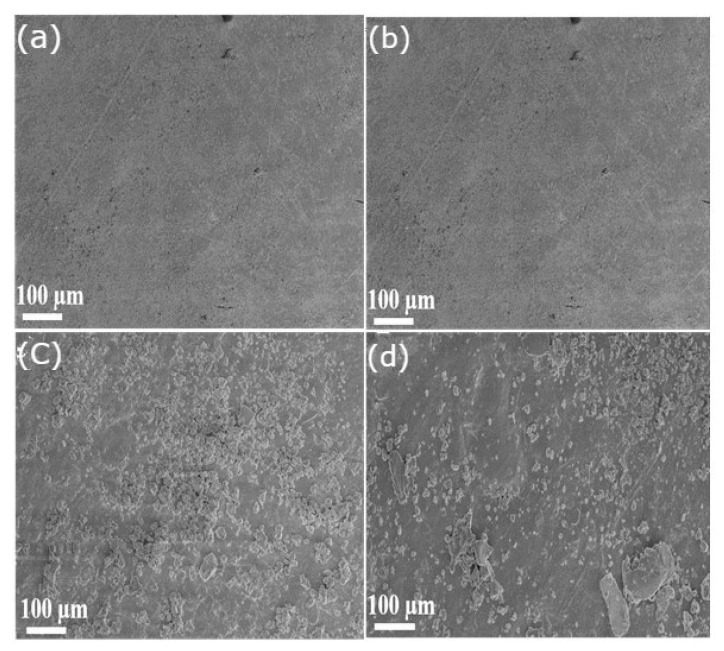
SEM images of the base (**a**) EPA and (**b**) EPA/mica 5 wt.%, (**c**) EPA/mica 10 wt.%, (**d**) EPA/mica 15 wt.%.

The morphological study conducted on the epoxy acrylate resin, with varying concentrations of mica nanoparticles ranging from initial to final additions of less than 20%, revealed substantial differences in the surface structure and dispersion of the composite material. SEM images, as depicted in Figure 11, vividly illustrate the impact of mica addition on the resin matrix. The incorporation of mica nanoparticles led to a notably more homogeneous dispersion within the resin, resulting in improved interfacial adhesion between the epoxy acrylate resin and the mica particles. This enhanced adhesion is indicative of a finer and more uniform morphology observed in the modified resin compared to the virgin resin, underscoring the better compatibility between mica and the epoxy acrylate matrix.

Furthermore, the morphological alterations observed signify the development of a more structurally homogeneous composite material with the addition of mica. This enhanced homogeneity suggests potential improvements in the material’s strength and durability. By shedding light on the microstructural changes induced by mica, the morphological investigation offers valuable insights into the intricate interaction between the filler and the resin matrix. Understanding these interactions is crucial for the development of high-performance materials tailored to specific applications, particularly in industries where mechanical strength and structural integrity are paramount.

**Figure 12:**
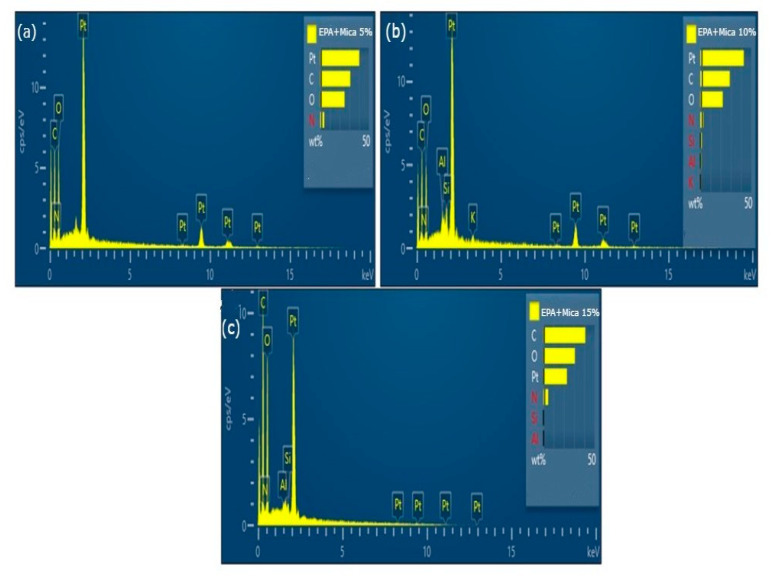
EDS images of (**a**) 5% EPA/mica, (**b**) 10% EPA/mica, and (**c**) 15% EPA/mica.

Elemental analyses of the initial and final epoxy acrylate resins with 5, 10, and 15 wt.% mica fillers revealed detectable differences in the elemental compositions of the composite materials, as shown in Figure 12. Energy Dispersive X-ray Spectroscopy (EDS) results demonstrated the presence of mica components within the modified resin, indicating the successful dispersion and integration of the filler material. Elemental mapping further illustrated a more uniform distribution of elements throughout the resin matrix following the addition of mica, highlighting increased homogeneity. Notably, the elemental analyses showed altered elemental ratios in the changed resin, particularly with the presence of Al, Si, and K, indicating interactions between the mica and resin elements. These elemental alterations provide valuable insights into the compositional variance induced by mica, offering crucial information for understanding the chemical compatibility and reinforcing effects of mica in epoxy acrylate resins. Such elemental analysis is essential for tailoring material characteristics to specific applications and gaining a deeper understanding of the synergistic effects between the resin and filler components.

**Figure 13:**
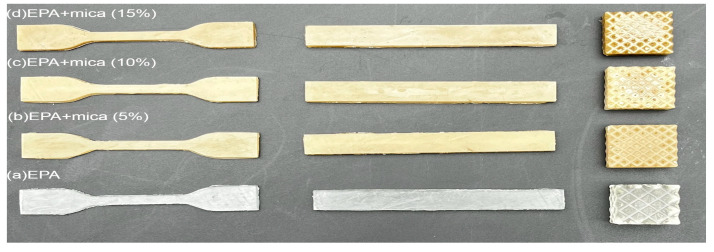
The 3D-printed samples images of (**a**) EPA, (**b**) 5% EPA/mica, (**c**) 10% EPA/mica, (**d**) 15% EPA/mica.

Figure 13 illustrates the various EPA+mica (5, 10, and 15) % concentrations in 3D-printed parts. The addition of 10% mica to epoxy acrylate resin has resulted in major advancements in 3D printing applications, thereby increasing the capabilities and performances of the printed structures. The addition of mica improves the mechanical strength and thermal stability of the resin, making it ideal for high-performance 3D printing applications. Increased flexural and tensile strengths aid the creation of stronger and longer-lasting 3D-printed items. Furthermore, the homogeneity obtained through morphological enhancements, as seen in scanning electron microscopy, guarantees that mica is evenly distributed inside. These improvements to the epoxy acrylate resin with mica make it a promising material for 3D printing in industries that require long-lasting, high-strength components, such as aerospace, automotive, and electronics, demonstrating the potential of mica-modified resins to advance the capabilities and applications of 3D printing technology. The resin matrix improves the overall print quality [27].

## 4. Conclusions

In summary, the incorporation of 10% mica into epoxy acrylate resin resulted in significant and beneficial alterations to the characteristics of the material. Through comprehensive structural analyses employing techniques such as FTIR and XRD, significant changes were observed at both the molecular and crystalline levels, indicating potential interactions between mica and the resin matrix. SEM imaging further demonstrated enhanced mica homogeneity and distribution within the resin, contributing to the observed increase in material strength. Elemental analysis confirmed the successful integration of mica particles into the resin, leading to alterations in composition and elemental ratios. Functional changes observed in FTIR spectra, such as shifts in peaks associated with acrylic acid and epoxy functionalities, further supported the notion of mica–resin interactions. Additionally, heat gravimetric analysis indicated improved thermal stability. Importantly, mechanical testing revealed a remarkable 132% increase in flexural strength and an 85% increase in tensile strength, positioning the material as a promising candidate for various applications, particularly in 3D printing, where robust mechanical and thermal properties are essential. Future studies should focus on refining formulations tailored for specific industrial needs and optimizing mica content to achieve the desired combination of properties. Overall, this comprehensive investigation provides valuable insights and paves the way for further advancements and applications of mica-modified epoxy acrylate resins in cutting-edge materials and manufacturing processes.

## Figures and Tables

**Table 1:** DLP 3D Printer Experiments.

Layer Height:	0.1 [mm]/0.05 [mm]	Bottom Lift	3 [mm]
Bottom layer count	4 [layers]	Lifting Distance:	3 [mm]
Exposure time	3 [s]	Bottom Lift Speed:	120 [mm/min]
Bottom exposure Time	8.5 [s]	Lifting Speed:	120 [mm/min]
Light-off delay	4 [s]	Retract Speed:	180 [mm/min]
Bottom light-off delay	4 [s]		

**Table 2:** Different formulations of resins and mica filler concentrations.

Sr	Sample Name	EPA	EPA/Mica5	EPA/Mica10	EPA/Mica15
1	CN991 wt.%	15	15	14	14
2	CN104NS wt.%	30	28	25	25
3	ACMO wt.%	25	22	22	20
4	TPGDA(SR306) wt.%	5	5	5	5
5	TMPTA(SR454) wt.%	17.5	17.5	15.5	12.5
6	PETA(SR444) wt.%	5	5	5	5
7	TPO wt.%	2.5	2.5	2.5	2.5
8	Mica wt.%	-	5	10	15

**Table 3:** Mechanical properties of EPA and EPA/mica composites.

Sr.	Sample Name	Tensile Strength (Mpa)	Tensile Elongation (%)	Density (g/cc)	Flexural Strength (Mpa)	Flexural Modulus (Mpa)
1	EPA	11.48	1.94	1.1847	8.8426	439.48
2	EPA/mica5 wt.%	20.41	2.04	1.2123	12.4363	616.98
3	EPA/mica10 wt.%	21.20	2.10	1.2762	20.4796	1010.83
4	EPA/mica15 wt.%	10.57	2.37	1.3257	12.9881	531.91

## Data Availability

Data are contained within this article.
